# Administration of a Probiotic Can Change Drug Pharmacokinetics: Effect of *E. coli* Nissle 1917 on Amidarone Absorption in Rats

**DOI:** 10.1371/journal.pone.0087150

**Published:** 2014-02-05

**Authors:** Zuzana Matuskova, Eva Anzenbacherova, Rostislav Vecera, Helena Tlaskalova-Hogenova, Milan Kolar, Pavel Anzenbacher

**Affiliations:** 1 Department of Pharmacology and Institute of Molecular and Translational Medicine, Faculty of Medicine and Dentistry, Palacky University Olomouc, Olomouc, Czech Republic; 2 Department of Medical Chemistry and Biochemistry, Faculty of Medicine and Dentistry, Palacky University Olomouc, Olomouc, Czech Republic; 3 Institute of Microbiology, Academy of Sciences of the Czech Republic, Prague, Czech Republic; 4 Department of Microbiology, Faculty of Medicine and Dentistry, Palacky University Olomouc, Olomouc, Czech Republic; Biological Research Centre of the Hungarian Academy of Sciences, Hungary

## Abstract

The growing interest in the composition and effects of microbiota raised the question how drug pharmacokinetics could be influenced by concomitant application of probiotics. The aim of this study was to find whether probiotic *E. coli* strain Nissle 1917 (EcN) influences the pharmacokinetics of concomitantly taken antiarrhythmic drug amiodarone (AMI). Live bacterial suspension of probiotic EcN (or non-probiotic *E. coli* strain ATCC 25922) was applied orally to male Wistar rats for seven days, while a control group of rats was treated with a saline solution. On the eighth day, the amiodarone hydrochloride was administered as one single oral dose (50 mg/kg) to all rats (N = 60). After 0, 1, 2, 3, 4, 5.5, 7, 9, 14, 22, and 30 hours, blood samples were taken from the rat abdominal aorta. The plasma level of AMI and its metabolite N-desethylamiodarone (DEA) was determined using the HPLC with UV detection. Administration of EcN led to a 43% increase of AMI AUC_0-30_ in comparison with control samples. However, this effect was not observed if EcN was replaced by a reference non-probiotic *E. coli* strain. Thus, EcN administration was most probably responsible for better drug absorption from the gastrointestinal tract. Plasma levels of DEA were also increased in plasma samples from animals treated with EcN. This change was again not found in the experiment with the reference non-probiotic strain. Higher DEA levels in samples from EcN-treated rats may be explained either by better absorption of AMI and/or by an increased activity of CYP2C forms, known to participate in metabolism of this drug, after EcN administration. In this paper, it is documented that concomitantly taken probiotic EcN may modulate pharmacokinetics of a drug; in this case, it led to an increased bioavailability of AMI.

## Introduction

Most of epithelial surfaces of human body such as the skin and the mucosa are colonized by a vast number of microorganisms, which are collectively known as microbiota or microbiome. Our microbiota contains trillions of bacterial cells and most of them never cultivated by classical methods, and represents a complex ecosystem with enormous microbiota diversity. Molecular biological methods had allowed revolutionary advance in microbiological research: the components of the human microbiota started to be analysed and identified. Our microbiome is producing enormous quantity of molecules able to interact with the host; however, the role of these molecules remains to be elucidated. Molecular analytical methods bring every day new pieces of knowledge about the major bacterial groups present in various body compartments, their changes during ontogeny, and their alterations in patients with organ and systemic disease when compared with healthy subjects, as well as other features of the microbiota. The largest number of bacteria resides in the digestive tract, with the highest density in the colon. Bacteria present in the intestine participate in nutrition and metabolism processes. The effect of the microbiota on the macroorganism started to be elucidated in a number of functional studies [1]. The role of microbiota and the function of mucosal barrier in maintaining human health were recently appreciated. There is a growing interest concerning the role of microbiota in etiopathogenesis of inflammatory and neoplastic diseases [2]. However, the studies concentrated on the microbiota influence on drug pharmacokinetics are still very sparse.

Based on the growing evidence of the importance of microbiota for health, the efforts to affect the composition of microbiota in an optimal direction are gathering momentum. In recent years, there has been considerable progress in understanding the mechanisms of probiotic action and in the future this should help to select suitable bacterial strains which could beneficially affect mucosal barrier function, immune responses, and suppression of inflammation [3]. The knowledge of the effects of simultaneous administration of drugs and probiotics on drug pharmacokinetics is still very limited. The complexity of mechanisms by which the fate of orally administered drugs could be affected by probiotics is discussed in recently published comprehensive review Stojančevic et al., 2013 [4]. The importance of both, human and commensal microbiota components in drug efficacy and toxicity was recently documented and pointed out Haiser et al., 2013 [5].

The aim of our study was to analyse the effect of probiotic bacteria applied orally on a drug (amidarone) pharmacokinetics in a rat model. The gram-negative bacterium of *Escherichia coli* Nissle 1917 of serotype O6:K5:H1 (EcN) is a fecal isolate with a lipopolysaccharide (LPS) consisting of a bisphosphorylated hexaacyl lipid A and a tetradecasaccharide containing one *E. coli* O6 antigen repeating unit. EcN was shown to have immunomodulating properties without showing immunotoxic effects [6], [7]. It has been used as a probiotic agent in medicine for the treatment or prevention of intestinal disorders and diseases since the early 1920s [8] and is commercially available [9]. For example, EcN can be used in treatment of diverticulosis, non-ulcer dyspepsia, antibiotic – associated colitis, intestinal mycoses, chronic constipation, inflammatory bowel disease, protracted or chronic recurrent diarrhea, or, primarily, in the treatment of irritable bowel syndrome [10]. The administration of EcN is safe and well tolerated [11].

On the other hand, the unlimited use of probiotics may lead to unwanted side-effects [12]. Thus, a question arises whether these microorganisms are safe when a drug is taken. For example, it is not known if the probiotic EcN (and other probiotics as well) can affect the pharmacokinetics of concomitantly taken drugs. In this paper, the antiarrhythmic drug amiodarone (AMI) was used to study whether the probiotic EcN can affect AMI pharmacokinetics. AMI is a drug used for treatment of ventricular tachycardia and ventricular fibrillation [13], [14] and is metabolized by cytochrome P450 enzymes (CYPs) [15], [16]. N-desethylamiodarone (DEA) is its main, less active metabolite. Because of its long half-life (on an average 58 day) [17], AMI organ toxicity is potentially more severe and difficult to manage than toxic reactions of other drugs with shorter half-lives [18]. The combination of AMI with probiotics like EcN could, in principle, influence its pharmacokinetics and hence become another factor influencing bioavailability of AMI.

The current work belongs to first studies dealing with the potential influence of probiotic bacteria on pharmacokinetics of a drug. In 2008, studies were published showing changes of gliclazide pharmacokinetics in diabetic rats pre-treated by a mixture of three probiotics (*L. acidophilus*, *L. rhamnosus* and *Bifidobacterium lactis*) in suspension prepared from freeze-dried probiotic powders mixed with HPLC water (Al Salami et al. [19]). They have found that in presence of probiotics, the biodistribution of gliclazide in rats was suppressed; however, in the diabetic animals, the effect was just the opposite [19]. The authors suggested an alteration of regulation of the mucosal transporting systems [20]. In the most recent literature, there is only a note on increased azoreductase activity during concomitant administration of sulfasalazine (SSZ) and mixture of three probiotic bacteria [21]; however, pharmacokinetic parameters of the drug as well as of its metabolite were not significantly different from control rats given SSZ alone. Also, according to Kunes et al. [22], the pharmacokinetics of 5-aminosalicylic acid in rat was not significantly changed by EcN medication compared to control animals. Our current studies demonstrate an increase in bioavailability of a drug (AMI) after premedication of rats with probiotic EcN bacteria, documented here for the first time.

## Materials and Methods

### Chemicals

All reagents and chemicals were purchased from Sigma-Aldrich CZ (Prague, Czech Republic), except for sodium chloride and EDTA used for preparation of the saline solution or for sampling, respectively, which were obtained from Lach-Ner (Neratovice, Czech Republic).

### Ethics Statement

The experiment was carried out in accordance with the Act No. 359/2012 Coll. on the protection of animals against abuse. All procedures with animals were approved by the Ethics Committee, Ministry of Education, Czech Republic.

### Study design and sampling

Live bacterial suspension of probiotic *Escherichia coli* Nissle 1917 in a phosphate buffered saline was applied intragastrically (0.7×10^9^ CFU/dose) to male Wistar rats (body weight 230–258 g, average weight 254 g, nine weeks old). The probiotic suspension was administered daily to thirty animals for seven days. Another group of thirty rats (body weight 222–258 g, average weight 250 g, nine weeks old) were stressed by oral application of a saline solution daily for seven days as well. This group was used as the control. On the eighth day, the suspension of amiodarone hydrochloride in water was applied as one single oral dose (50 mg/kg) to all rats (N = 60). After 0, 1, 2, 3, 4, 5.5, 7, 9, 14, 22, and 30 hours, blood samples were taken from the rat abdominal aorta. The reference, non-pathogenic but non-probiotic strain of *E. coli* ATCC 25922, was also used in this study. This strain of *E. coli* was administered also intragastrically (0.7×10^9^ CFU/dose) to thirty male Wistar rats (body weight 314–354 g, average weight 320 g, ten weeks old). This group was compared with another group of thirty rats (body weight 296–360 g, average weight 312 g, ten weeks old) stressed by oral application of the saline solution. The experiment with this reference strain was performed using the same protocol as described above for the *E. coli* Nissle 1917 strain. Subsequently, 7 ml blood samples with 0.2 mol/L EDTA were centrifuged and the plasma samples were frozen at −70°C.

### Preparation of biological samples

In a 1.5 ml Eppendorf tube (Sarstedt, Nümbrecht, Germany), 100 µL of the plasma sample was mixed with 5 µL of an internal standard solution of 0.02 mmol/L trifluoperazine dihydrochloride. After deproteinization by the addition of 300 µl acetonitrile, the mixture was centrifuged at 18 400× g at 4°C for 5 min. The supernatant was carefully transferred to a clean test tube and evaporated under nitrogen at 40°C. The residue in the test tube was dissolved in a 100 µL of the methanol∶ water (1∶1) mixture. Thirty µL of this prepared sample was then analyzed by HPLC.

### Preparation of standards

AMI and DEA were dissolved in the mixture of acetonitrile∶ water (1∶1). Plasma standards were prepared by the addition of an appropriate volume of AMI and DEA to drug-free plasma to obtain final concentrations of 0.1 to 1.6 µg/mL of AMI, and 0.02 to 0.4 µg/mL of DEA. The stock solution of the internal standard trifluoperazine dihydrochloride was prepared by dissolving in water to a final concentration of 0.02 mmol/L; 5 µL of this solution was added to each sample. Control samples for determination of the intra-day and inter-day determinations of precision and accuracy were prepared by the addition of AMI and DEA to drug-free plasma to obtain concentrations of 0.1, 0.4, and 0.8 µg/mL of AMI and 0.05, 0.1, and 0.4 µg/mL of DEA, respectively. All samples were stored at −70°C.

### HPLC conditions

HPLC-UV analyses were performed on the Ultimate 3000 system (Dionex, Sunnyvale, California, USA). The plasma levels of AMI and DEA were obtained using a Kinetex PFP column (150×4.6 mm ID) with a 2.6 µm particle size (Phenomenex, Torrance, California, USA) protected by a Security Guard (4×2 mm ID) precolumn with a C18 reverse phase of the same origin. The mobile phase for separation of AMI, DEA, and internal standard (trifluoperazine) consisted of 24 mmol/L acetic acid with 8.2 mmol/L triethylamine∶ methanol∶ acetonitrile (3∶6∶16 (v/v)) delivered at a flow-rate of 1 mL/min at 40°C. The typical run time was 12 min. The separated components were detected by UV detector at 242 nm [23]. The internal standard was used for the construction of AMI and DEA calibration curves. For calibration, the following ranges of concentration levels were chosen: AMI, 0.1, 0.2, 0.3, 0.4, 0.5, 0.6, 0.8 and 1.6 µg/mL, and DEA, 0.02, 0.05, 0.07, 0.10, 0.2, 0.3 and 0.40 µg/mL. Linear regression gave the values of the coefficient of determination r^2^ = 0.9961 for AMI, and r^2^ = 0.9990 for DEA. The average recovery for AMI and for its metabolite DEA was 75% and 82%, respectively. The coefficient of variation of the precision and the accuracy determination (intraday and interday) was less than 15%. The limit of AMI and DEA quantitation was determined as 0.050 µg/mL and 0.018 µg/mL, respectively, established as a peak signal to noise of baseline ratio equivalent to 10∶1. Data collection, integration, and calibration were accomplished using the Chromeleon Chromatography Data System Version 6.80 (Dionex, Sunnyvale, California, USA).

## Results

**Figure 1:**
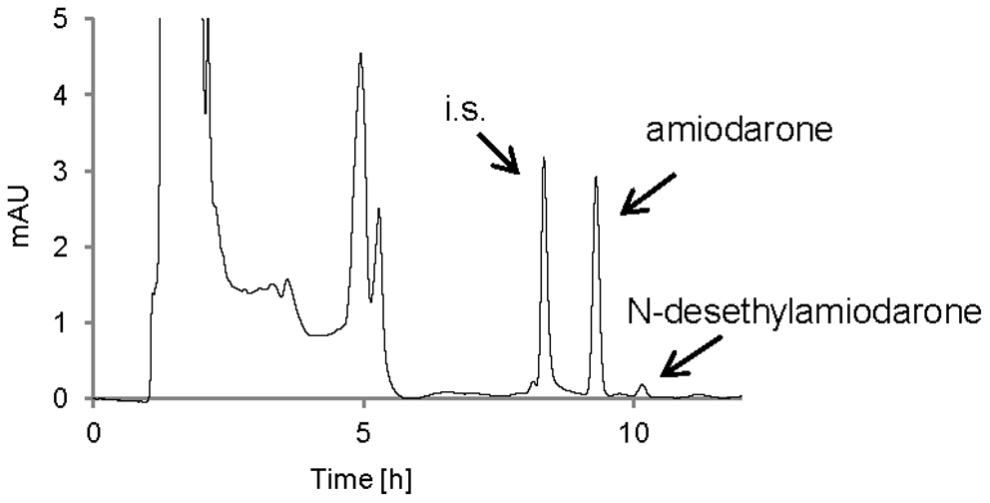
Chromatographic profile of three compounds in biological sample: trifluoperazine (an internal), amiodarone, N-desethylamiodarone. Legend Fig. 1: i.s.: an internal standard trifluoperazine (8.32 min); amiodarone (9.31 min); N-desethylamiodarone (10.11 min). The HPLC chromatogram was obtained from the rat blood plasma sample taken 3 hours after amiodarone application.

**Figure 2:**
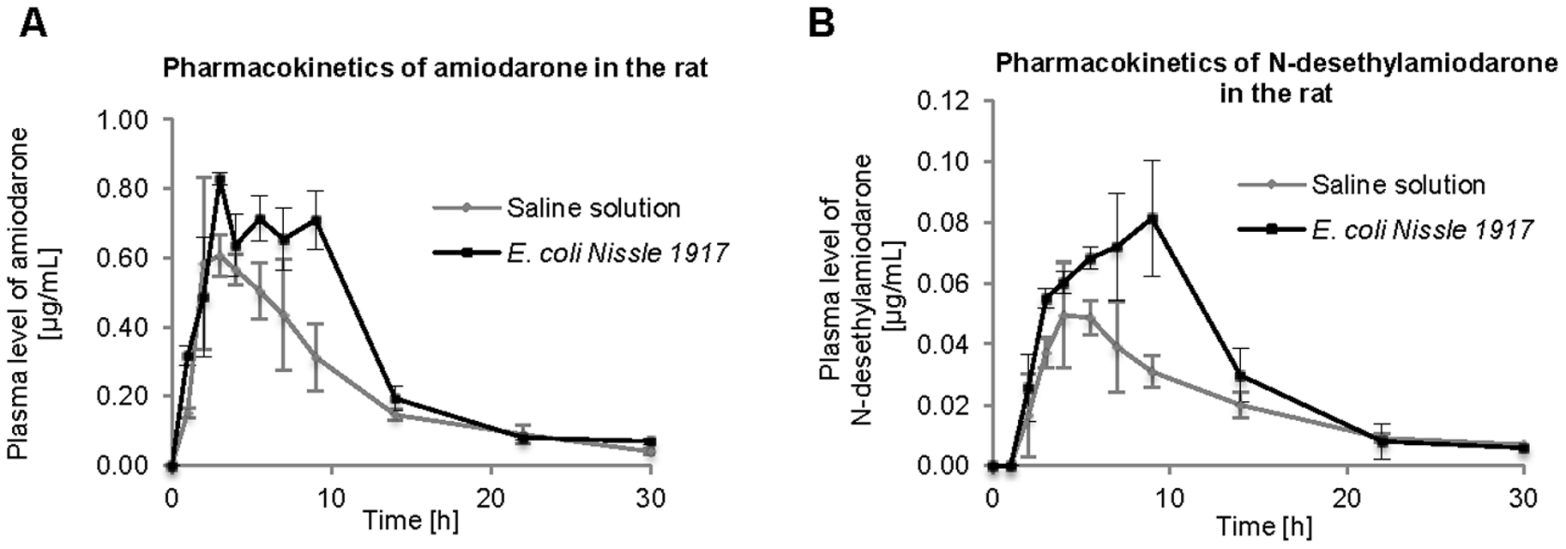
Influence of *E. coli* Nissle 1917 on the pharmacokinetics of amiodarone (A) and N-desethylamiodarone (B). Legend Fig. 2A: Pharmacokinetics of amiodarone with or without (control group) probiotic *E. coli* Nissle 1917 pre-treatment. Each point is presented as means ± S.D.; N = 3. Legend Fig. 2B: Pharmacokinetics of N-desethylamiodarone (metabolite of amiodarone) with or without (control group) probiotic *E. coli* Nissle 1917 pre-treatment. Each point is presented as means ± S.D.; N = 3.

**Figure 3:**
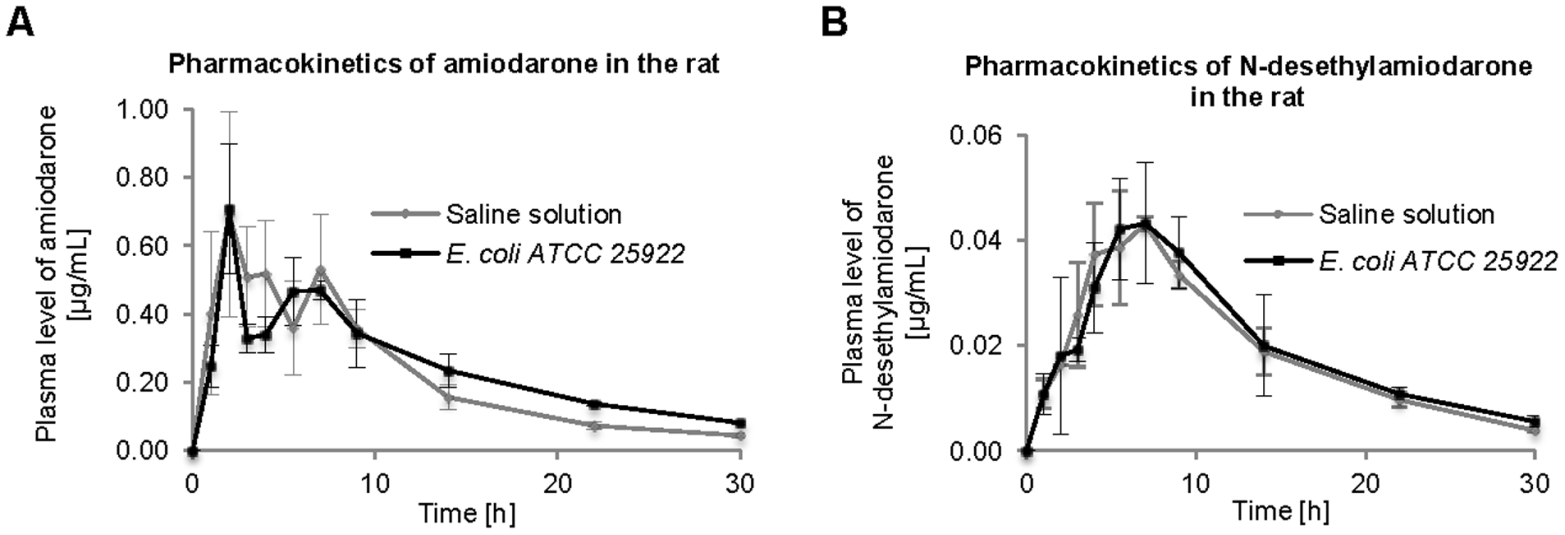
Influence of a non-probiotic bacteria on the pharmacokinetics of amiodarone (A) and N-desethylamiodarone (B). Legend Fig. 3A: Pharmacokinetics of amiodarone with or without (control group) non-probiotic *E. coli* ATCC 25922 pre-treatment. Each point is presented as means ± S.D.; N = 3. Legend Fig. 3B: Pharmacokinetics of N-desethylamiodarone (metabolite of amiodarone) with or without (control group) non-probiotic *E. coli* ATCC 25922 pre-treatment. Each point is presented as means ± S.D.; N = 3.

Figure 1 shows a typical HPLC chromatogram to document the separation of AMI, DEA, and trifluoperazine (an internal standard). Amiodarone pharmacokinetics was obtained from both EcN probiotic-premedicated experimental animals as well as animals with the administration of the non-probiotic *E. coli* strain. Results of both of these experiments, i.e. two types of AMI pharmacokinetics, were compared to data obtained in a control experiment when no bacteria were administered to experimental animals, just the AMI. Fig. 2A shows the experimental data for AMI and Fig. 2B for its main metabolite DEA after EcN premedication, while Figs. 3A and 3B document the time course of the AMI and DEA pharmacokinetics obtained with a non-probiotic strain administration. Based on these pharmacokinetics data, it can be seen that the administration of probiotic EcN bacteria has led to increased bioavailability of the drug.

The pharmacokinetic parameters (i.e. the time required to reduce the maximal level of a drug (c_max_) to one half, t_1/2_; time needed to reach the maximal level of the respective drug t_max_; area under the curve for 30 hours, i.e. time of the experiment, AUC_0-30_; or the AUC extrapolated to infinity, AUC_0-∞_) were derived from the plasma level *vs.* time curve for AMI and DEA and are shown in Table S1 and Table S2. The increased plasma levels of AMI were observed after EcN administration to rats (AUC_0-30_ = 9.31±0.65 h.µg/mL) in comparison with samples from the control rats (AUC_0-30_ = 6.52±0.60 h.µg/mL). On the other hand, the administration of reference non-probiotic strain of *E. coli* ATCC 25922 did not markedly affect the pharmacokinetics of AMI in the rat (AUC_0-30_ = 7.37±0.53 h.µg/mL) in comparison with the application of saline solution to the control rats (AUC_0-30_ = 6.74±0.56 h.µg/mL).

In the case of amiodarone metabolite DEA, the peak concentration of DEA after application of EcN to rats (Fig. 2B) was higher (c_max_ = 0.09±0.01 µg/mL) and shifted by more than 2.5 hours in comparison with control samples (c_max_ = 0.06±0.01 µg/mL). Its peak concentration, as well as the pharmacokinetic plasma level *vs.* time course, was not markedly changed after the administration of reference strain of *E. coli* ATCC 25922 in comparison with control samples, as documented in Fig. 3B.

## Discussion

The experiments discussed here were performed *in vivo* with experimental animals as models to assess the influence of probiotic bacteria on the drug pharmacokinetics. As demonstrated by our results (Figs. 2A, 2B), the pharmacokinetics of AMI and its metabolite DEA in animals treated with probiotic *E.coli* bacteria markedly differ from the data obtained from control animals. The AUC_0-30_ of AMI was 1.4 times higher in the rat plasma from EcN-treated animals in comparison with the treatment using saline solution (a control). Also, the pharmacokinetics of its main metabolite DEA exhibited a different time course, with maximum levels shifted by more than 2.5 hours to a longer time interval; moreover, the AUC_0-30_ was 1.6 times higher in EcN-treated animals.

Interestingly, these changes in drug pharmacokinetics were not observed with the non-probiotic strain of the *E. coli* bacteria (ATCC 25922). In this case, the pharmacokinetics of both the AMI as well as its main metabolite did not markedly differ (Figs. 3A, 3B).

The reasons for increased bioavailability of orally applied AMI due to administration of the probiotic EcN are difficult to explain as there are several simultaneously occurring phenomena which are based on the properties and local effects of the probiotic as well as the drug. Moreover, the current knowledge about the detailed mechanisms of EcN action and effects as well as about the regulation of expression and function of the corresponding proteins is scattered and obtained usually *in vitro* and under various conditions being thus only an approximation of processes occurring *in vivo*. Hence, the attempts to elucidate the effects obtained in this work remain to a great extent speculative. Nevertheless, on the basis of data known from the literature it is possible to delineate two ways which may suggest an explanation.

First, there is a decrease of local pH in the intestine due to the presence of EcN [24]. This effect has been attributed to production of short chain fatty acids. Amiodarone, a weak base (pKa of AMI is 8.7 at 37°C [25], [26]), is then better ionized in lower pH which may facilitate its movement across the mucosal layer and finally its disposition. Intestinal mucosal layer is known to be a barrier to lipophilic drugs and the ionization of a drug may have a positive effect on its diffusion through mucus [27]. Another attempt to explain better disposition of amiodarone may be based on an increased expression of the Oatp2B1 (Slco2B1) transporter known to mediate the influx of amiodarone in the intestinal cells [28]. The expression of this transporter was shown to be regulated by levels of proinflammatory cytokine TNF-alpha [29]. It may be speculated that in response to lowered levels of this cytokine in presence of EcN (for a review, see [30]), the expression of the Oatp2B1 transporter may be in turn higher leading to better bioavailability of this drug. In fact, also the changes in gliclazide permeation in diabetic and normal rats after premedication with probiotics were ascribed to changes in regulation of mucosal transporting systems [20].

AMI is metabolized to the main active metabolite DEA by CYP1A1, 1A2, 2C8, 2C19, 2D6, and 3A4 enzymes in humans and by CYP1A1, 1A2, 2C6, 2C11, 2D1, 2D2, and 3A1 enzymes in rats [15], [16]. Following the administration of AMI in the presence of EcN, increased plasma levels of DEA were observed in comparison with control samples. The higher c_max_ value and its shift to longer time intervals (approximately by 3 h) are probably caused by a better disposition of AMI, which is discussed above. Moreover, moderately increased activity of the liver CYP2C forms after administration of EcN found in our earlier study [31] may contribute to increased levels of the DEA. These changes were observed again only in the experiment with EcN administration while no changes were found in the experiment with the reference non-probiotic *E. coli* strain ATCC 25922 application.

In conclusion, this study shows an effect of administration of a probiotic strain, here the *Escherichia coli* Nissle 1917, on the pharmacokinetics of amiodarone in rats. Increased drug absorption caused by this probiotic can be the result of the interplay of various factors influencing regulation of transport systems in the intestine including metabolic reactions and changes in intestinal microbiota composition [4], [32]. Interestingly, on the contrary, the non-probiotic strain of these bacteria does not possess the properties leading to a better bioavailability of amiodarone. In conclusion, concomitantly taken probiotic EcN may modulate the pharmacokinetics of AMI as well as its metabolite DEA by increasing the bioavailability of this drug. The changes caused by the probiotic are probably not so prominent that they are likely to be important in clinical use except perhaps for the time delay in reaching the concentration maximum. It should be mentioned here that the results obtained cannot be directly extrapolated to other drugs or probiotic bacteria due to apparent complexity of the processes in the intestine. In other words, there may be similar or even greater effects observed with other drugs and microbiota or, in other cases, no influence of probiotics on pharmacokinetics of a drug may be seen. In the case of AMI and EcN, on the basis of results described here it can be reasonably expected that the simultaneous uptake of the EcN strain and this drug would most probably pose no harm to the patient.

## Supporting Information

Table S1 **Pharmacokinetic (PK) parameters in rats after oral administration of amiodarone (50 mg/kg) with or without (control group) probiotic** ***E. coli*** **Nissle 1917 pre-treatment.** Legend Table S1: AMI: amiodarone; DEA: N-desethylamiodarone; t_1/2_: half-life; c_max_: maximum drug concentration; t_max_: time to reach c_max_; AUC: area under the curve. Results are expressed as mean ± S.D., N = 3. Values of parameters significantly differing from controls are in bold.
(DOC)

Table S2 **Pharmacokinetic parameters in rats after oral administration of amiodarone (50 mg/kg) with or without (control group) non-probiotic** ***E. coli*** **ATCC 25922 pre-treatment.** Legend Table S2: AMI: amiodarone; DEA: N-desethylamiodarone; t_1/2_: half-life; c_max_: maximum drug concentration; t_max_: time to reach c_max_; AUC: area under the curve. Results are expressed as mean ± S.D., N = 3.
(DOC)
