# Supplementary material for: Administration of a Probiotic Can Change Drug Pharmacokinetics: Effect of E. coli Nissle 1917 on Amidarone Absorption in Rats
Source: PLoS One. 2014 Feb 5;9(2):e87150. doi: 10.1371/journal.pone.0087150 (PMC3914806; doi:10.1371/journal.pone.0087150)
Supplement: Table S1 — Pharmacokinetic (PK) parameters in rats after oral administration of amiodarone (50 mg/kg) with or without (control group) probiotic E. coli Nissle 1917 pre-treatment. Legend Table S1: AMI: amiodarone; DEA: N-desethylamiodarone; t1/2: half-life; cmax: maximum drug concentration; tmax: time to reach cmax; AUC: area under the curve. Results are expressed as mean ± S.D., N = 3. Values of parameters significantly differing from controls are in bold. (DOC) [file pone.0087150.s001.doc]

**Table S1. Pharmacokinetic (PK) parameters in rats after oral administration of amiodarone (50 mg/kg) with or without (control group) probiotic *E. coli*** Nissle 1917 pre-treatment.

| **Determined compound** | **AMI** | | **DEA** | |
| --- | --- | --- | --- | --- |
| **Application** | ***E. coli* Nissle 1917** | **Saline solution** | ***E. coli* Nissle 1917** | **Saline solution** |
| t_1/2_ [h] | 11.19 ± 1.34 | 8.63 ± 0.91 | 6.92 ± 1.93 | 11.38 ± 2.76 |
| c_max_ [µg/mL] | 0.82 ± 0.02 | 0.78 ± 0.16 | 0.09 ± 0.01 | 0.06 ± 0.01 |
| t_max_ [h] | 3.83 ± 1.18 | 3.50 ± 1.47 | **8.33 ± 0.94** | 5.50 ± 1.22 |
| AUC_0-30h_ [h^.^µg/mL] | **9.31 ± 0.65** | 6.52 ± 0.60 | **0.97 ± 0.10** | 0.60 ± 0.03 |
| AUC_0-∞h_ [h^.^µg/mL] | **10.47 ± 0.67** | 7.06 ± 0.50 | **1.03 ± 0.08** | 0.72 ± 0.05 |

AMI: amiodarone; DEA: N-desethylamiodarone; t_1/2_: half-life; c_max_: maximum drug concentration; t_max_: time to reach c_max_; AUC: area under the curve. Results are expressed as mean ± S.D., N = 3. Values of parameters significantly differing from controls are in bold.
