# Supplementary material for: Administration of a Probiotic Can Change Drug Pharmacokinetics: Effect of E. coli Nissle 1917 on Amidarone Absorption in Rats
Source: PLoS One. 2014 Feb 5;9(2):e87150. doi: 10.1371/journal.pone.0087150 (PMC3914806; doi:10.1371/journal.pone.0087150)
Supplement: Table S2 — Pharmacokinetic parameters in rats after oral administration of amiodarone (50 mg/kg) with or without (control group) non-probiotic E. coli ATCC 25922 pre-treatment. Legend Table S2: AMI: amiodarone; DEA: N-desethylamiodarone; t1/2: half-life; cmax: maximum drug concentration; tmax: time to reach cmax; AUC: area under the curve. Results are expressed as mean ± S.D., N = 3. (DOC) [file pone.0087150.s002.doc]

**Table S2. Pharmacokinetic parameters in rats after oral administration of amiodarone (50 mg/kg) with or without (control group) non-probiotic *E. coli*** ATCC 25922 pre-treatment.

| **Determined compound** | **AMI** | | **DEA** | |
| --- | --- | --- | --- | --- |
| **Application** | ***E. coli* ATCC 25922** | **Saline solution** | ***E. coli* ATCC 25922** | **Saline solution** |
| t_1/2_ [h] | 11.00 ± 2.71 | 7.08 ± 0.69 | 7.86 ± 0.33 | 7.32 ± 1.31 |
| c_max_ [µg/mL] | 0.66 ± 0.16 | 0.84 ± 0.18 | 0.05 ± 0.01 | 0.04 ± 0.01 |
| t_max_ [h] | 3.17 ± 1.65 | 3.67 ± 2.36 | 6.50 ± 0.70 | 6.00 ± 1.40 |
| AUC_0-30h_ [h^.^µg/mL] | 7.37 ± 0.53 | 6.74 ± 0.56 | 0.60 ± 0.09 | 0.54 ± 0.01 |
| AUC_0-∞h_ [h^.^µg/mL] | 8.70 ± 0.23 | 7.27 ± 0.50 | 0.66 ± 0.11 | 0.59 ± 0.02 |

AMI: amiodarone; DEA: N-desethylamiodarone; t_1/2_: half-life; c_max_: maximum drug concentration; t_max_: time to reach c_max_; AUC: area under the curve. Results are expressed as mean ± S.D., N = 3.
